# Reconfiguration Error Correction Model for an FBG Shape Sensor Based on the Sparrow Search Algorithm

**DOI:** 10.3390/s23167052

**Published:** 2023-08-09

**Authors:** Qiufeng Shang, Feng Liu

**Affiliations:** 1 Department of Electronic and Communication Engineering, North China Electric Power University, Baoding 071003, China; lindashqf@126.com; 2 Hebei Key Laboratory of Power Internet of Things Technology, Department of Electronic and Communication Engineering, North China Electric Power University, Baoding 071003, China; 3 Baoding Key Laboratory of Optical Fiber Sensing and Optical Communication Technology, Department of Electronic and Communication Engineering, North China Electric Power University, Baoding 071003, China

**Keywords:** shape sensor, FBG, error correction, curvature, optimization algorithm

## Abstract

A reconfiguration error correction model for an FBG shape sensor (FSS) is proposed. The model includes curvature, bending direction error correction, and the self-correction of the FBG placement angle and calibration error based on an improved sparrow search algorithm (SSA). SSA could automatically correct the placement angle and calibration direction of the FBG, and then use the corrected placement angle and calibration direction to correct the curvature and bending direction of the FSS, thereby improving the accuracy of shape reconfiguration. After error correction, the tail point reconfiguration errors of different shapes were reduced from 2.56% and 4.96% to 1.12% and 2.45%, respectively. This paper provides a new reconfiguration error correction method for FSS that does not require a complicated experimental calibration process, is simpler, more efficient, and more operable than traditional methods, and has great potential in FSS application scenarios.

## 1. Introduction

In recent years, the fiber Bragg grating (FBG) shape sensor (FSS) has been extensively researched in the field of optical fiber sensing. This technology has several advantages, such as compact structure, high flexibility, resistance to harsh environments and corrosion, and reusability, compared with other shape reconfiguration technologies. The FSS has immense application potential in civil, mechanical, aerospace, biological, medical, and other fields [1,2,3,4,5,6].

An FSS constructed from multiple single-mode optical fibers is one of the most common fiber optic shape sensors. The Frenet–Serret spatial differential geometry reconfiguration algorithm, based on curvature measurement, is the most widely applicable shape reconfiguration method. The basic principle is as follows: the curvature and bending direction at different positions are calculated by measuring central wavelength shifts in different cores at specific cross-sectional positions, and the shape is reconstructed through numerical integration combined with specific algorithms [7]. There is usually some error in the measurement of curvature and bending direction, and improving measurement accuracy for FBG curvature and bending direction is an important part of the FBG shape sensing field.

In order to improve the accuracy of FBG curvature measurement, researchers have conducted extensive research on the sensitivity of FBG curvature measurement [8,9]. For an FBG multi-core shape sensor, the placement angle deviation and calibration direction error of the FBG are the main factors that lead to the measurement error of curvature and bending direction [10,11,12]. The superposition of the two seriously affects curvature and bending direction measurement accuracy and may even cause incorrect shape reconfiguration results. Therefore, it is an important matter in the field of FBG shape sensing to study correction methods for curvature and bending direction errors in an FSS.

Various error correction methods have been widely investigated to overcome the influence of FBG placement angle error and calibration error on FSS reconfiguration accuracy. Kim et al. used a calibration matrix to correct the curvature error in an FBG flexible shape sensor by repeatedly calibrating the same curvature and taking the average value [13]. This method has a large randomness level and does not consider the effect of FBG placement angle error; moreover, the compensation effect is not obvious. Lou et al. quantitatively analyzed the manufacturing and calibration process of FBG shape sensors and determined the influence of the placement angle and calibration error for each FBG on the curvature measurement error [11]. This error correction process is cumbersome and complex, and the error accuracy of the experimental equipment is mainly analyzed through manual methods, resulting in greater subjectivity and limitations. Lv et al. reduced curvature measurement error by substituting the curvature correction coefficient into the curvature calibration relationship equation and using shape reconfiguration error as an evaluation index to correct the equation. This method ignores the influence of the FBG placement angle on the measured curvature [14]. Tan et al. proposed a self-calibration method based on a genetic algorithm to correct the placement angle in FSS, which automatically calibrates the placement angle of the sensor according to the measured strain and angle, resulting in an improved measurement accuracy of the curvature and bending direction [12]. The influence of the calibration direction deviation is ignored, and the need for accurate strain data increases the complexity of the experiment. The measurement error correction of FSS curvature and bending direction is a complex and difficult process. Existing methods have problems such as high experimental complexity, poor experimental repeatability, a small range of applications, and lack of strict theoretical model support.

In this study, we established an FSS reconfiguration error delivery model based on the Frenet–Serret framework and error delivery theory; a simulation experiment was conducted to verify the method. In addition, in view of the problem that FBG placement angle deviation and calibration error are difficult to determine, this paper uses an improved SSA to automatically correct the placement angle deviation and calibration error, and establishes an FSS reconfiguration error correction model based on the optimization of the improved SSA. The experiment verified the feasibility and practicability of the model. The results show that the reconfiguration error correction model can improve measurement accuracy of curvature and bending direction, and effectively enhance the reconfiguration accuracy in FSS. The proposed method is simple, innovative, and convenient to use, with wide applicability. Owing to its significantly superior performance over existing methods, it has immense potential for use in practical scenarios requiring FSS.

## 2. FBG Shape Sensing Error Model

Curvature measurement is the basis of FBG shape measurement. Obviously, a small-er spatial resolution and higher curvature measurement accuracy can result in a more accurate shape reconfiguration. The interpolation method is applied to eliminate the influence of spatial resolution on reconfiguration accuracy [15]. The interpolation process is based on the measured curvature of the detection points. FBG calibration deviation and placement angle deviation directly affect the measurement accuracy of the curvature, thereby affecting the accuracy of the interpolation results; they may also lead to incorrect shape reconfiguration results. Presently, few studies have proposed reconfiguration error models for the FBG shape sensor. It is of great significance to establish a three-dimensional shape perception error model addressing the shape reconfiguration error, FBG calibration direction, and placement angle.

**Figure 1:**
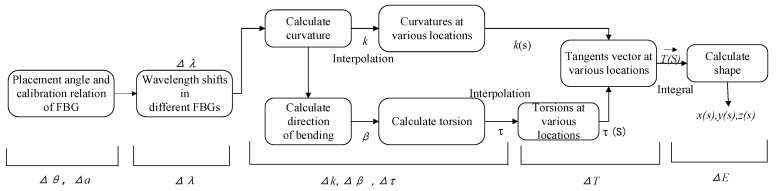
FSS error delivery model. The bending curvature *k* and bending direction *β* at the detection point can be obtained according to the curvature in each core *k_i_*, and the discrete local *k* and *β* are converted into the curvature and torsion functions *k*(s) and *τ*(s) through interpolation.

Shape reconfiguration algorithms based on the Frenet–Serret framework constitute the most widely applied method. The flow scheme and error delivery process of the shape reconfiguration algorithm are shown in Figure 1. The spatial coordinates *x*(s), *y*(s), and *z*(s) of the curve are obtained by solving the Frenet equation between the tangent vector $\vec{T\left(s\right)}$, normal vector, and subnormal vector of the curvature point [16].

The error delivery process of the shape reconfiguration algorithm based on the Frenet–Serret framework is as follows: the calibration direction deviation and placement angle deviation of the FBG cause the measurement curvature error Δ*k* and bending direction error Δ*β*, resulting in the calculation errors of the torsion, Δ*τ*, and tangent vector, Δ*T*. Δ*T* is accumulated during the integration process, eventually resulting in reconfiguration errors.

### 2.1. Curvature Error and Bending Direction Error Correction Model

**Figure 2:**
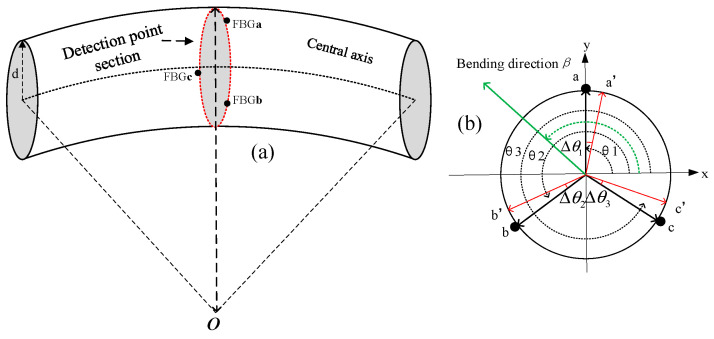
(**a**) Illustration of cross-section at the detection point. (**b**) Placement angle deviation diagram.

For a three-core fiber-optic shape sensor with a symmetrical distribution of the FBGs and a mutual angle of 120°, the shape sensor cross-section at the detection points is schematically shown in Figure 2. In Figure 2a, FBG**a**, FBG**b**, and FBG**c** represent the three FBGs of the detection point, and d represents the cross-sectional radius of the shape sensor.

The angle of the line connecting each FBG at the detection point to the neutral axis of the sensor relative to the x-axis is set to *θ_i_*, the sensing curvature of each FBG is *k_i_*, and is orthogonally decomposed, the curvature and bending direction at the detection point can be expressed as Equations (1)–(3).

(1) $$\vec{k(s)}=\sum_{i=1}^{3}k_{i}\cos\theta_{i}\vec{x}-\sum_{i=1}^{3}k_{i}\sin\theta_{i}\vec{y}$$

(2) $$k=\frac{2\left|\vec{k(s)}\right|}{3}=\frac{2\sqrt{{\left(\sum_{i=1}^{3}k_{i}\cos\theta_{i}\right)}^{2}+{\left(\sum_{i=1}^{3}k_{i}\sin\theta_{i}\right)}^{2}}}{3}$$

(3) $$\beta =\arctan\left(\frac{k_{y}}{k_{x}}\right)=\arctan\left(\frac{\sum_{i=1}^{3}k_{i}\sin\theta_{i}}{\sum_{i=1}^{3}k_{i}\cos\theta_{i}}\right)$$

where $\vec{x}\;\mathrm{and}\;\vec{y}$ are unit vectors along the x-axis and y-axis, respectively, and $\vec{k\left(s\right)}$ is the sum of the curvature vectors at point s, which is used to solve the curvature function.

In this paper, when the FBG is uniformly distributed as *θ*_1_ = 90°, *θ*_2_ = 210°, and *θ*_3_ = 330°, the expressions of the curvature and bending direction can be simplified as follows:

(4) $$k=\frac{1}{3}\sqrt{{\left(2k_{1}-k_{2}-k_{3}\right)}^{2}+3{\left(k_{2}-k_{3}\right)}^{2}}$$

(5) $$\beta =\arctan\left(\frac{2k_{1}-k_{2}-k_{3}}{\sqrt{3}\left(k_{2}-k_{3}\right)}\right)$$

It is difficult to ensure that the FBG is evenly distributed on the surface of the substrate at equal angles during the manufacturing of the shape sensors; there is usually a certain placement angle deviation in the placement position of the FBG. As shown in Figure 2b, a, b, and c represent the ideal placement positions for FBGs. Assuming that the placement of the angle errors of FBGs is Δ*θ_i_*, the actual placement positions of the FBG is a′, b′, and c′. The FBGs no longer satisfy the uniform placement condition, and solving for the curvature and bending direction in Equations (4) and (5) would introduce placement angle deviations, leading to curvature and bending direction measurement errors. 

In this paper, a compensation model for curvature errors and bending direction errors is proposed to solve the above problems. When there is a deviation in the laying angle of the FBG, the curvature and bending direction can still be calculated using Equations (2) and (3). Substitute the laying angle deviation of FBG into Equations (2) and (3) to obtain Equations (6) and (7).

(6) $$k=\frac{2\left|\vec{k(s)}\right|}{3}=\frac{2\sqrt{{\left(\sum_{i=1}^{3}k_{i}\cos\left(\theta_{i}+\Delta \theta_{i}\right)\right)}^{2}+{\left(\sum_{i=1}^{3}k_{i}\sin\left(\theta_{i}+\Delta \theta_{i}\right)\right)}^{2}}}{3}$$

(7) $$\beta =\arctan\left(\frac{k_{y}}{k_{x}}\right)=\arctan\left(\frac{\sum_{i=1}^{3}k_{i}\sin\left(\theta_{i}+\Delta \theta_{i}\right)}{\sum_{i=1}^{3}k_{i}\cos\left(\theta_{i}+\Delta \theta_{i}\right)}\right)$$

After the shape sensor is manufactured, the scale factor *H* between the measured curvature *k* and the central wavelength shift Δ*λ* for each FBG should be calibrated. The relationship between *k* and Δ*λ* can be expressed as Equation (8). where *η* is the strain transfer coefficient, *P_e_* is the elastic coefficient, *λ*_B_ is the FBG central wavelength, and *d* is the cross-sectional radius of the shape sensor.

(8) $$k=\frac{2\eta \Delta \lambda }{\lambda_{B}d(1-P_{e})}=H\times \Delta \lambda$$

**Figure 3:**
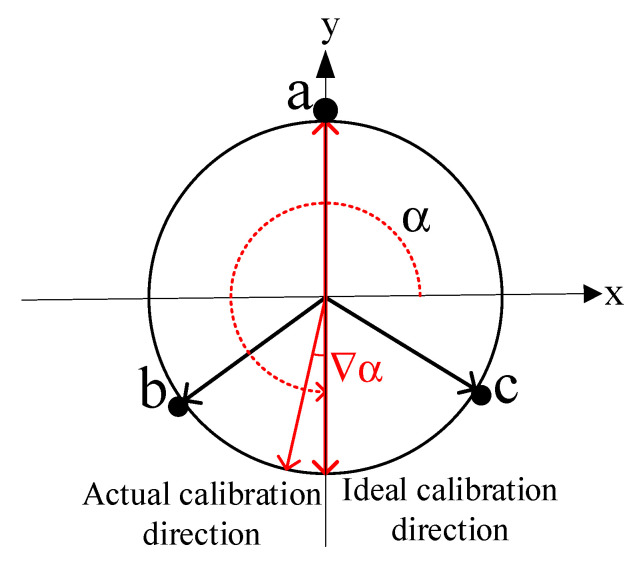
Schematic diagram of calibration direction deviation.

During the calibration process, the FBG should be placed in the direction where the curvature of bending is maximal, that is the calibration direction α, but an angular deviation usually occurs during the actual calibration. Assuming that the FBG calibration direction deviation is Δα, this is illustrated in Figure 3.

According to the geometric relationship of the points on the cross-section, the curvature relationship under the calibration error is shown in Equation (9):

(9) $$k=\frac{2\eta \Delta \lambda }{\lambda_{B}d(1-P_{e})\cdot \cos\Delta \alpha }=\frac{H\cdot \Delta \lambda }{\cos\Delta \alpha }$$

By substituting Equation (9) into Equations (6) and (7), the error correction model for the curvature and bending direction measurement at the shape sensor detection point is obtained, as shown in Equations (10) and (11):

(10) $$k=\frac{2\left|\vec{k(s)}\right|}{3}=\frac{2\sqrt{{\left(\sum_{i=1}^{3}\left(k_{i}/\cos\Delta \alpha_{i}\right)\cdot \cos\left(\theta_{i}+\Delta \theta_{i}\right)\right)}^{2}+{\left(\sum_{i=1}^{3}\left(k_{i}/\cos\Delta \alpha_{i}\right)\cdot \sin\left(\theta_{i}+\Delta \theta_{i}\right)\right)}^{2}}}{3}$$

(11) $$\beta =\arctan\left(\frac{k_{y}}{k_{x}}\right)=\arctan\left(\frac{\sum_{i=1}^{3}\left(k_{i}/\cos\Delta \alpha_{i}\right)\cdot \sin\left(\theta_{i}+\Delta \theta_{i}\right)}{\sum_{i=1}^{3}\left(k_{i}/\cos\Delta \alpha_{i}\right)\cdot \cos\left(\theta_{i}+\Delta \theta_{i}\right)}\right)$$

Given the placement error angle of each FBG, calibration direction error, and FBG measurement curvature, by substituting these into Equations (10) and (11), the curvature error and bending direction error at the detection point can be corrected.

### 2.2. Error Correction Model Verification and Analysis

**Figure 4:**
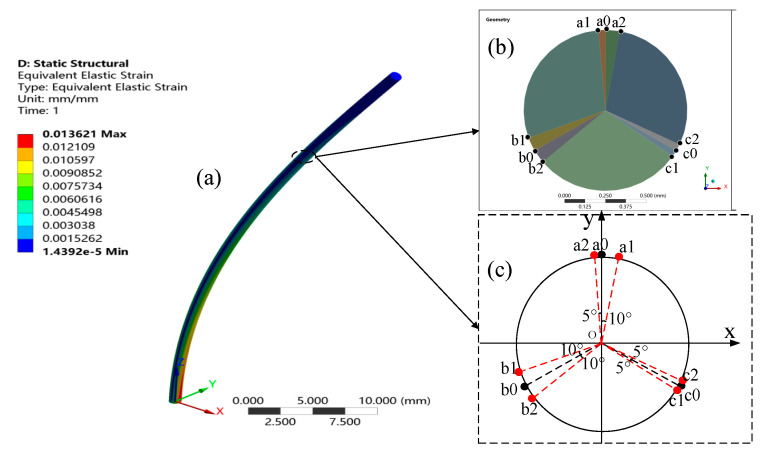
(**a**) Shape sensor simulation model. (**b**) Simulation model cross-section. (**c**) FBG placement diagram.

To verify the feasibility and validity of the error correction models in Equations (10) and (11) for the curvature and bending direction, a finite element simulation model of the FBG multicore fiber-optic shape sensor was developed in this paper using ANSYS Workbench software. As shown in Figure 3, the sensor has a cross-sectional radius r of 0.5 mm, length of 50 mm, and spatial resolution of 0.25 mm. The dynamic finite element model of the FBG was established at nine different positions on the sensor surface to simulate the actual placement of the FBG. Figure 4b shows the cross-section at one of the sensor’s detection points, and Figure 4c shows the specific placement angles of the FBG at the detection points.

The shape of the simulated model was changed by applying a mechanical load to it to obtain a specific curvature at each point of the cross-section, where the sensor bends in the direction. The detection data at each point were divided into three groups; the data at the points a0, b0, and c0 are Data1, the data at points a1, b1, and c1 are Data2, and the data at points a2, b2, and c2 are Data3. a0, b0, and c0 are set to be uniformly distributed at 120° in the cross-section and are considered the ideal placement angle for the FBG. With a0 as the reference point, as shown in Figure 3b, b1, b2, c1, and c2 indicate the placement of FBG**b** and FBG**c** under different placement angle deviations. a0 is in the direction of the greatest bending force and can be used as the ideal calibration direction; hence, a1 and a2 represent the detection data of FBG**a** under different calibration direction errors.

**Figure 5:**
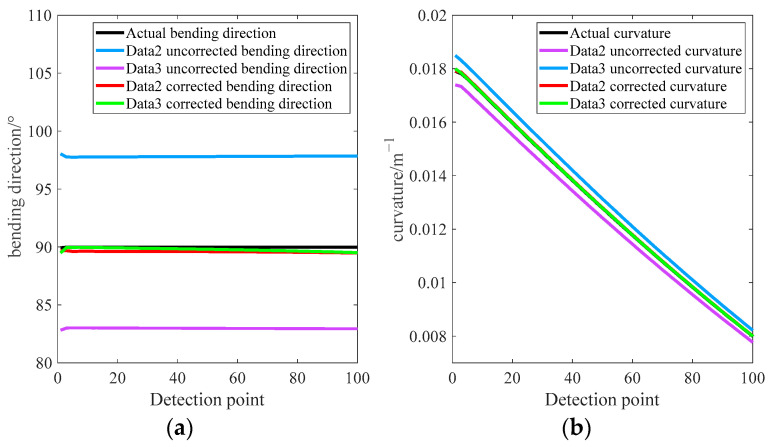
Calculation of curvature and bending direction obtained by different methods. (**a**) Bending direction calculation results. (**b**) Bending curvature calculation results.

Different calibration direction errors and placement angle errors are introduced into Data2 and Data3, which are, respectively, substituted into Equations (4) and (5) as well as our error correction model to solve the curvature and bending direction of the model. The experimental results are shown in Figure 5.

It can be seen from Figure 5 that the curvature and bending direction of Data2 and Data3 calculated using Equations (4) and (5) have obvious errors, which are determined by the magnitude of the FBG placement angle deviation and calibration direction deviation. After correcting the curvature and bending direction errors using our error correction model, the measured curvature and bending direction at each detection point of the simulation model becomes consistent with the actual situation, which establishes the feasibility and effectiveness of the error correction model proposed in this paper.

**Figure 6:**
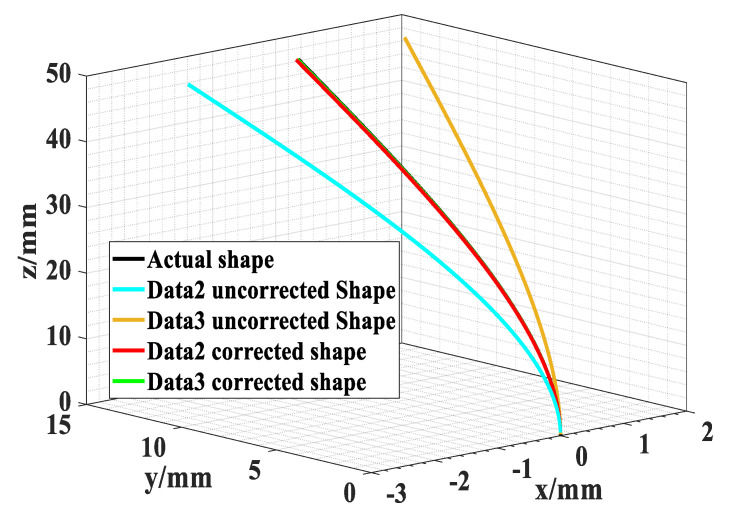
Shape reconfiguration results of different data groups.

Three sets of data were used to reconstruct the shape of the model. The reconstructed shape with Data1 was used as the actual shape of the model. The results of the model shape reconfiguration under different data groups are shown in Figure 6. According to the shape reconfiguration algorithm, the shape reconfiguration error reaches the maximum at the tail points. The reconfiguration error of the optical fiber shape sensor is usually expressed by the Euclidean distance error ∆E, as shown in Equation (12) [17,18,19,20,21,22].

(12) $$\Delta E=\sqrt{{\left(x-x_{0}\right)}^{2}+{\left(y-y_{0}\right)}^{2}+{\left(z-z_{0}\right)}^{2}}$$

In Equation (12), *x*, *y*, and *z* are the reconstructed coordinates of the detection point, and *x*_0_, *y*_0_, and *z*_0_ are the actual coordinates of the detection point. In this study, we used the tail point reconfiguration error *φ*_max_ and maximum relative error *R*_max_ (ratio of *φ*_max_ to the model length) as the evaluation indicators to compare the shape reconfiguration results.

According to the experimental results in Figure 6, the shape reconfiguration errors *φ*_max_ of the method in Equations (4) and (5) were 1.40 mm and 1.42 mm, for which the *R*_max_ values were 2.8% and 2.84%, respectively. The shape reconfiguration errors of the errors *φ*_max_ of the correction model were 0.39 mm and 0.27 mm, for which the *R*_max_ values were 0.78% and 0.54%, respectively.

Next, the bending direction of the sensor model was changed, and the datasets for Data2 and Data3, collected under different bending directions, were used to reconstruct the shape. The reconfiguration results before and after the error correction were compared and analyzed, and the results are shown in Table 1.

The relative errors of Data2 and Data3 calculated using Equations (4) and (5) were 2.082% and 1.894%, respectively, which were reduced to 0.315% and 0.308%, respectively, after the model correction. It was verified that the error correction model proposed in this paper could correct the measured curvature and bending direction under different calibration direction deviations and placement angle deviations and improve the shape reconfiguration accuracy of the sensor.

## 3. Experiment

### 3.1. Experimental System and Sensor Calibration

**Figure 7:**
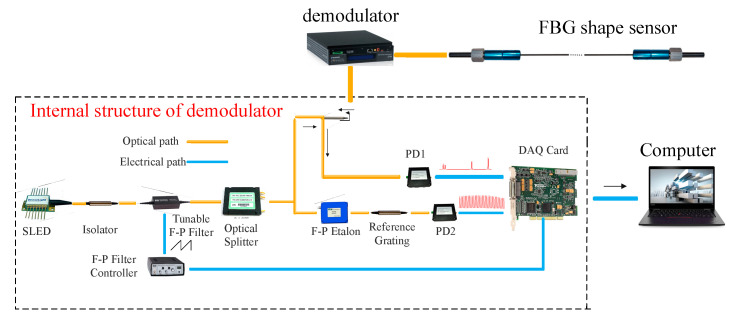
Experimental sensing system.

We constructed an FBG shape sensing system as shown in Figure 7. The whole system consists of an FBG shape sensor, a demodulator (resolution of 0.1 pm), and a computer. The main components of the demodulator include a broadband light source, an isolator, a Fabry–Perot (FP) filter, optical coupler, circulator, an FP etalon, a photodetector, and a data acquisition card. When demodulating the spectrum, the optical coupler divides the narrow-band adjustable light into two branches; the upper branch is transmitted to the FBG sensor through the circulator, the reflected light is detected by the photodetector 1 (PD1), and the lower branch is detected by the photodetector 2 (PD2). PD1 detects the maximum light intensity when the transmitted wavelength of the FP filter coincides with the reflected wavelength of the FBG. The detected light signal was converted by PD1 and PD2 into an electrical signal, which was transmitted via a data acquisition card to a personal computer for subsequent signal processing.

**Figure 8:**
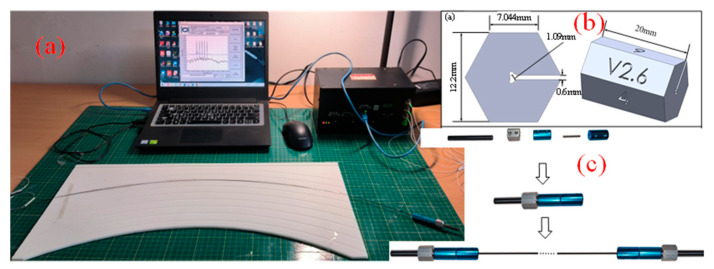
(**a**) System diagram for the calibration experiment. (**b**) FBG fixtures. (**c**) Calibration tools.

In order to verify the practicability of the error correction model, this paper fabricated the FSS through the custom-made FBG. The FSS consisted of three FBG arrays with placement on the surface of the nickel–titanium alloy wire. The epoxy resin is used as the adhesive to fix the FBG array on the surface of the nickel–titanium alloy wire. The nickel–titanium alloy wire has an effective length of 465 mm and a diameter of 1 mm. Each FBG array is inscribed with 5 FBGs at equal intervals, the diameter of the optical fiber is 375.2 μm, the length of the FBG is 13 mm, and the spacing of the adjacent gratings is 100 mm. As shown in Figure 8a, a calibration plate was produced by 3D printing, and multiple arcs with fixed curvature were inscribed on the surface of the calibration plate, and the FSS was embedded in the groove for curvature calibration. FBG fixtures and calibration tools were designed using 3D printing to fix the placement angle and calibration direction of FBG, as shown in Figure 8b,c.

**Figure 9:**
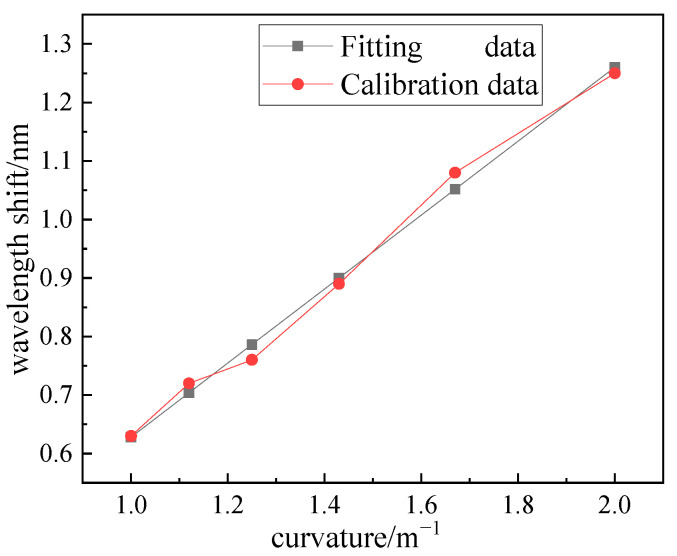
Calibration result.

Rotate the calibration tools so that the FBG is in the direction of maximum force to obtain the FBG central wavelength shift data under different curvatures, and perform least-square fitting on the discrete data to obtain the scale factor *H*. The calibration results are shown in Figure 9, and the fitted linear relationship expression is *k* = 1.26∆*λ* + 0.21(nm/m^−1^).

### 3.2. Optimization Model

According to the error correction process, we know that to correct the reconfiguration error of the shape sensor, we need to obtain the FBG placement angle deviation Δ*θ_i_* and the calibration direction deviation Δ*α_i_* at each detection point of the sensor. This paper proposes a method based on an improved SSA to determine Δ*θ_i_* and Δ*α_i_*. SSA is a new swarm intelligence optimization algorithm proposed by Xue et al. [23]. Compared with other swarm intelligence optimization algorithms, it has the characteristics of a high search accuracy, fast convergence speed, good stability, and strong robustness [24].

The foraging process of sparrows can be abstracted as a discoverer–joiner model, and a reconnaissance and early warning mechanism is added. The discoverer guides the population to search and forage, which has the advantages of high adaptability and a wide search range. The joiner follows the finder to forage for better fitness. At the same time, some joiners will monitor the finders to increase their own predation rate. However, SSA, like other swarm intelligence optimization algorithms, has the problem that it is easy to fall into the local optimum [25]. We introduce the Chebyshev chaos map into the SSA algorithm to initialize the population distribution and improve the uniform distribution of the population space. Chebyshev map is a typical representative of chaotic maps. Compared with other chaotic maps, it has better chaotic characteristics, a wider value range, and a faster convergence speed [26]. Its expression is as follows:

(13) $$\left\{ \begin{array}{ll} x_{t+1}=\cos[t\cdot \arccos\left(x_{t}\right)], & -1\leq x_{t}\leq 1\\ y_{t}=x_{t+1}, & t=0,1,2,\ldots ,n\\ y_{t+1}=\left(2/\pi \right)\times \arctan\left[\cos\left(y_{t}\right)\right]+M, & -1\leq x_{t}\leq 1 \end{array}\right.$$

**Figure 10:**
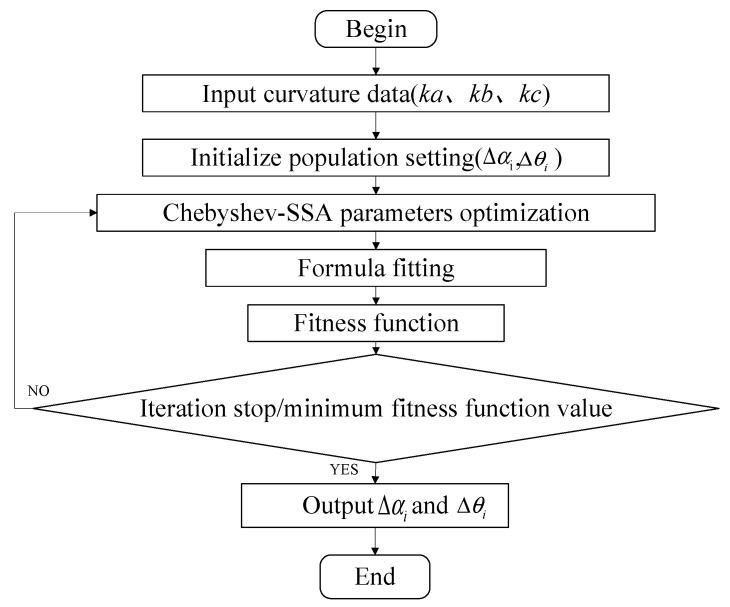
Flowchart of the optimization model.

In Equation (13), *y_t_*_+ 1_ is the sparrow individual after the *t* + 1th mapping iteration, and *M* is an arbitrary constant. The shape sensor bending curvature was set to *k_true_* during the calibration, and Δ*θ_i_* and Δ*α_i_* of each detection-point FBG are substituted into Equation (10) as the optimization parameters to obtain the calculated curvature *k* under different parameters. Let the measurement curvature error function be the fitness function, as shown in Equation (14). When Δ*k* reaches the minimum value, the optimization parameter is the actual deviation angle. The detailed process is shown in Figure 10.

(14) $$\Delta k=\left|k-k_{true}\right|$$

1. Enter the measured curvatures *k_a_*, *k_b_*, and *k_c_* for FBG a, FBG b, and FBG c in the fixed curvature state, respectively.
2. Initialize the inputs and set the placement angles *θ*_1_, *θ*_2_, and *θ*_3_ of the FBG sensor. In this paper, *θ*_1_ = 90°, *θ*_2_ = 210°, and *θ*_3_ = 330°.
3. Set the FBG calibration direction deviation Δ*θ_i_* and placement angle deviation Δ*α_i_*, and Δ*θ_i_* and Δ*α_i_* are randomly assigned and coded within a certain range.
4. Δ*θ_i_* and Δ*α_i_* are substituted into Equation (10) to obtain the theoretical curvatures *k*_1_, k_2_, and *k*_3_, and are optimized using Chebyshev-SSA.
5. The optimal parameters of Δ*θ_i_* and Δ*α_i_* are output when the value of the fitness function ∆*k* is minimized or at the end of the iteration. Otherwise, steps (3) to (5) are repeated.

**Figure 11:**
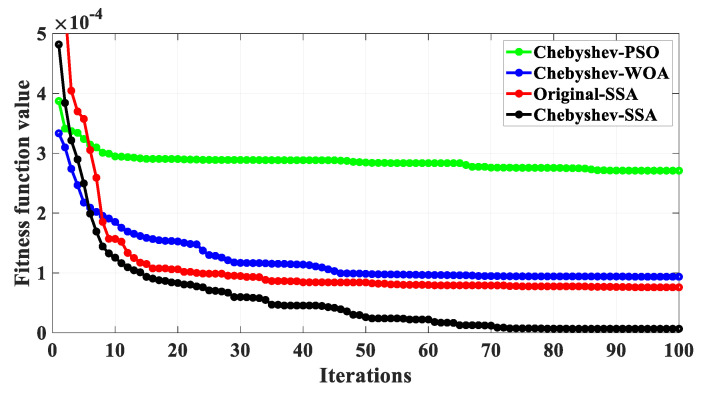
Iterative curves of different optimization algorithms.

In order to evaluate the performance of the Chebyshev-SSA optimization algorithm, it is compared with the original SSA, Chebyshev-PSO algorithm, and Chebyshev-WOA. Taking the calibration curvature *k* = 1/7 m^−1^ as an example, the above optimization algorithm is used to automatically correct the placement angle and calibration coefficient of the FBG, and the average value of the error iteration curves of different optimization models is obtained by repeating 10 times, as shown in Figure 11. It can be seen from Figure 11 that the convergence speed and convergence accuracy of the Chebyshev-SSA optimization algorithm are the best.

The calibration direction deviation and placement angle deviation of the FBG for each detection point of the shape sensor, obtained after parameter optimization, are shown in Table 2.

### 3.3. Shape Reconfiguration

We experimentally verified the effectiveness and practicability of the error correction model. Selecting a reasonable reconfiguration shape in the experiment is important. Any complex shape can be regarded as a composition of multiple micro-arc segments with constant curvature and torsion. As long as the error correction model is effective for shapes with a fixed curvature and torsion, it is also applicable to arbitrary complex shapes [27].

**Figure 12:**
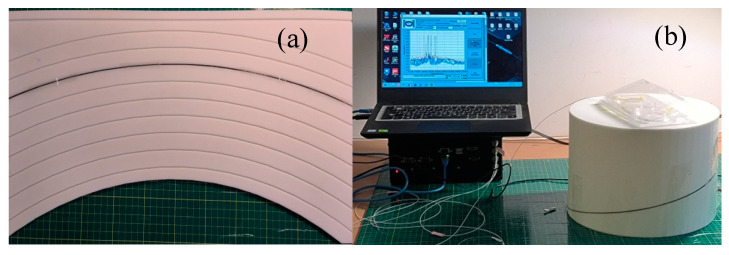
Experimental diagram for shape reconfigurations. (**a**) Arc shape reconfiguration. (**b**) Spiral shape reconfiguration.

A series of molds was designed as shape carriers for the sensors by using a calibration board and 3D printing technology. Figure 9a has multiple arcs of known curvature inscribed in the calibration plate, and Figure 12b is a 3D model designed using SolidWorks with spiral-shaped recesses of known curvature and deflection inscribed on the surface of the model; the spiral-shaped recesses have a circumference of 900 mm. As shown in Figure 12a,b, the shape sensor is fixed in the groove of the calibration plate and the 3D model to carry out the shape reconfiguration experiment.

**Figure 13:**
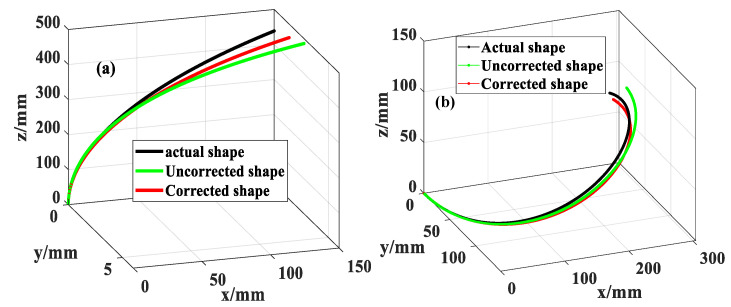
(**a**) Arc reconfiguration results. (**b**) Spiral reconfiguration results.

The error corrections for the curvature and bending direction were completed by substituting the deviations of the FBG calibration direction and deviations of the placement angle optimized by the Chebyshev-SSA model in Table 2 into Equations (10) and (11). The reconfiguration results before and after the corrections are compared and shown in Figure 13, with Figure 13a for 1/7 m^−1^ arc reconfiguration and Figure 13b for the spiral curve reconfiguration. The reconstructed shape of the sensor after error correction was closer to the actual shape. The tail point reconfiguration errors for the arc and spiral were 11.66 mm and 22.6 mm before the error correction, which were corrected to 5.23 mm and 11.4 mm, respectively. The average relative accuracy of shape reconfiguration is improved by 56.25% and 50.6%, respectively.

The reconfiguration experimental results for arcs of different curvatures are shown in Table 3. The experimental results show that the absolute reconfiguration errors of the tail points with arcs were reduced by 6.83 mm, and the relative errors were reduced by 1.47%.

## 4. Discussion

Based on the error model and error delivery theory of multicore fiber shape sensing under the Frenet–Serret framework, the error model for the relationship between the shape reconfiguration error, FBG calibration direction, and placement angle was established. The theoretical relationship between the bending curvature, bending direction, FBG calibration direction deviation, and placement angle deviation was derived, and an error-corrected model for the bending curvature and bending direction was proposed. The validity of the solution for correcting the curvature and bending direction was verified using a simulation model. The results show that the curvature and bending direction can be corrected by the method for different FBG calibration deviations and placement angle deviations.

In this paper, the Chebyshev mapping is introduced into SSA, and the errors correction model of the FBG calibration direction and placement angle based on Chebyshev-SSA optimization is established. Compared with the existing methods, the model has strict theoretical model support, can self-correct the calibration direction and placement angle of FBG during the sensor calibration process, and reduces the complexity of the experiment. Then, the optimization model was combined with the curvature and bending direction solution method proposed in this paper to construct the FBG shape sensor reconfiguration error correction model, and the experiment verified the feasibility and practicability of the error correction model. The results show that Chebyshev-SSA can simply and efficiently optimize the calibration direction and placement angle of FBG, and further improve the measurement accuracy of curvature and bending direction, thereby reducing the shape reconfiguration error of the FSS.

We propose a new method for correcting FBG calibration deviation and placement angle deviation. Compared with the existing methods, this method alleviates the limitations related to the accuracy of the experimental equipment and size of the sensor. In addition, it has strict theoretical model support and stronger applicability.

We perform reconfiguration experiments using shapes with fixed curvature and torsion. Because the complex shape will introduce more influencing factors [28,29], which is not conducive to the verification of the error correction model. In future research, we will study the influence of factors such as twist on the accuracy of FSS shape reconfiguration, and further improve the proposed reconfiguration error correction model.

## Figures and Tables

**Table 1:** Shape reconfiguration results under different bending directions.

Data Group		Data2					Data3				
**Bending direction/°**		80	90	200	210	330	80	90	200	210	330
***R*** **_max_** **/%**	**Uncorrected**	2.2	2.7	2.6	1.9	0.8	2.8	2.8	1.3	0.7	2.0
	**Corrected**	0.4	0.6	0.1	0.06	0.4	0.2	0.1	0.3	0.2	0.5

**Table 2:** Deviations of FBG calibration direction and placement angle at each test point.

Detection Point	Placement Angle Deviation/°			Calibration Direction Deviation/°		
	FBGa	FBGb	FBGc	FBGa	FBGb	FBGc
Point1	0	−3.9	7.5	3.9	12.5	2.2
Point2	7.5	−4.5	4.3	1.6	−8.0	1.8
Point3	2	5.3	−6.1	−9.2	8.4	−7.4
Point4	−4.1	10.5	5.8	11.2	−4.3	8.1
Point5	2.3	14.8	9.4	9.1	9.2	−3.5

**Table 3:** Results of different arc reconfigurations.

Radius of Curvature r/mm	Tail Point Reconfiguration Error			
	Uncorrected Error		Corrected Error	
	Absolute Error/mm	Relative Error/%	Absolute Error/mm	Relative Error/%
600	11.80	2.54	4.63	0.99
700	11.66	2.51	5.23	1.13
800	11.72	2.52	4.38	0.94
900	10.67	2.29	4.21	0.91
1000	12.50	2.69	5.65	1.22

## Data Availability

The data presented in this study are available on request from the corresponding author.
